# Exploring ND-011992, a quinazoline-type inhibitor targeting quinone reductases and quinol oxidases

**DOI:** 10.1038/s41598-023-39430-w

**Published:** 2023-07-28

**Authors:** Jan Kägi, Willough Sloan, Johannes Schimpf, Hamid R. Nasiri, Dana Lashley, Thorsten Friedrich

**Affiliations:** 1 Institut für Biochemie, Albert-Ludwigs-Universität Freiburg, Freiburg, Germany; 2 Department of Chemistry, William & Mary, Williamsburg, VA USA; 3 Department of Cellular Microbiology, University Hohenheim, Stuttgart, Germany

**Keywords:** Oxidoreductases, Membrane proteins, Target validation

## Abstract

Bacterial energy metabolism has become a promising target for next-generation tuberculosis chemotherapy. One strategy to hamper ATP production is to inhibit the respiratory oxidases. The respiratory chain of *Mycobacterium tuberculosis* comprises a cytochrome *bcc:aa*_*3*_ and a cytochrome *bd* ubiquinol oxidase that require a combined approach to block their activity. A quinazoline-type compound called ND-011992 has previously been reported to ineffectively inhibit *bd* oxidases, but to act bactericidal in combination with inhibitors of cytochrome *bcc:aa*_3_ oxidase. Due to the structural similarity of ND-011992 to quinazoline-type inhibitors of respiratory complex I, we suspected that this compound is also capable of blocking other respiratory chain complexes. Here, we synthesized ND-011992 and a bromine derivative to study their effect on the respiratory chain complexes of *Escherichia coli*. And indeed, ND-011992 was found to inhibit respiratory complex I and *bo*_3_ oxidase in addition to *bd*-I and *bd*-II oxidases. The IC_50_ values are all in the low micromolar range, with inhibition of complex I providing the lowest value with an IC_50_ of 0.12 µM. Thus, ND-011992 acts on both, quinone reductases and quinol oxidases and could be very well suited to regulate the activity of the entire respiratory chain.

## Introduction

Tuberculosis caused by *Mycobacterium tuberculosis* is one of the infectious diseases with the most causes of death worldwide mainly affecting developing countries^1^. With the emergence of COVID-19, tuberculosis was briefly replaced as the leading cause of death from a single infectious disease, but will again play a major role in the future. Since conventional medication is showing less and less success due to the development of multi-drug (MDR) and extensively drug-resistance (XDR)^2^, there is an urgent need for new medications. The *M. tuberculosis* enzyme complexes involved in energy metabolism that synthesise the vast majority of ATP have recently come into the focus of interest for the development of new antibiotics^3–5^. A major difficulty in controlling tuberculosis is that *M. tuberculosis* can enter a non-replicating phase^6^ when exposed to stresses, such as the lack of oxygen, and can persist in this phase for a very long time. Important for this survival mechanism are the enzyme complexes responsible for energy metabolism such as *bd* oxidase, which is up-regulated under these conditions^7^. The respiratory chain of *M. tuberculosis* comprises two terminal oxidases^8^. Under high oxygen tension the cytochrome *bcc:aa*_3_ oxidase is produced, while at low oxygen conditions the *bd* oxidase is active. Previous studies showed that inhibition of both *M. tuberculosis* terminal oxidases resulted in an effective growth suppression of antibiotic-tolerant mycobacteria^9^. Inhibition of cytochrome *bcc*:*aa*_3_ oxidase is achieved by the drug candidate Telacebec (Q203) that has already been tested in a phase 2a clinical efficacy trial^10^. To prevent *M. tuberculosis* from using the alternative route for electron transfer maintaining respiration and thus ATP synthesis^11^, the cytochrome *bd* oxidase has to be inhibited in combination with the cytochrome *bcc*:*aa*_3_ oxidase. Recently, a small organic compound N-(4-(4-(trifluoromethyl)phenoxy)phenyl)quinazolin-4-amine (also called ND-011992) was identified that was shown to inhibit cytochrome *bd* oxidase^9^. However, the potential use of ND-011992 as anti-tuberculosis drug is hampered by its poor pharmacokinetic properties as already mentioned in the original publication^9^. Thus, optimization of the lead structure is urgently needed to improve the inhibitory potential and pharmacokinetics of ND-011992^8,9^.

**Figure 1:**
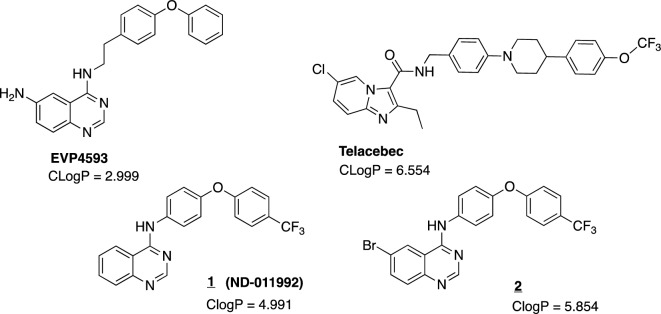
Structures and calculated partition coefficients (CLogP) of EVP4593, Telacebec, ND-011992 (**1**) and bromine derivative (**2**).

Furthermore, a similar quinazoline-based inhibitor 4-N-[2-(4-phenoxyphenyl)ethyl]-quinazolin-4,6-diamine (EVP4593) was reported by one of us to inhibit mitochondrial complex I, another important enzyme complex of the respiratory chain^12^. The common aromatic core structure of ND-011992 and EVP4593 opened up the question of whether ND-011992 is specific for *bd* oxidase or whether it could also inhibit complex I (Fig. 1).

In this work, the inhibition of complex I as well as the *bo*_3_ and *bd* terminal oxidases of the respiratory chain in *Escherichia coli* by ND-011992 and its bromine containing derivative, compound **2** (Fig. 1) were investigated. Compound **2** has an elevated clogP value, signifying heightened lipophilicity and a closer resemblance to the clogP of established drug candidate Telacebec when compared to compound **1**. Moreover, the motivation behind incorporating a halogenated derivative stemmed from the possibility that if compound **1** demonstrated good binding to one of the enzymes under investigation, the brominated compound **2** would be well suited to localize the binding site on this enzyme via cryo-electron microscopy. To this end, we are planning on conducting extensive structure–activity-relationship studies in our next work. The aforementioned enzymes are very well suited for this study, since the *E. coli bd* oxidases show a high structural similarity to the *M. tuberculosis bd* oxidase^8^. Furthermore, *E. coli* respiratory complex I is a structural minimal form of mitochondrial complex I. Our studies reveal that ND-011992 inhibits quinone reducing complex I and quinol oxidizing oxidases.

## Results

### Synthesis of ND-011992 and its derivative

**Figure 2:**
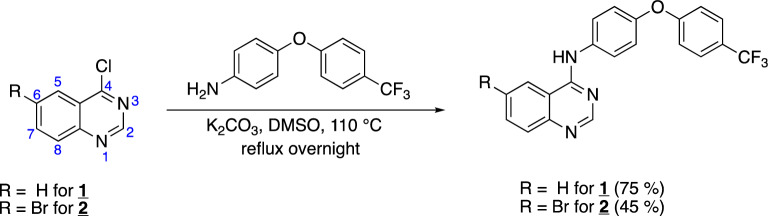
One-step synthesis of ND-011992 (**1**) and bromine derivative (**2**).

ND-011992 (**1**) and a ND-011992 derivative (**2**) with a bromine substituting for H at position 6 (Fig. 2) were synthesized in a one-step reaction modified from a procedure by Lee et al.^9^ as described in the ‘Methods’ section.

4-Chloroquinazoline or 6-Bromo-4-chloroquinazoline, respectively, were reacted with 4-(4-(trifluoromethyl)phenoxy)aniline and potassium carbonate in DMSO. All starting materials are commercially available and the reaction affords products in moderate yields (75% for **1** and 45% for **2**). Both crude compounds were purified via silica gel flash chromatography. Chlorine and iodine derivatives of this compound (not further described in this work) were synthesized in analogous manner. Compounds were characterized via ^1^H- and ^13^C-NMR as well as via MS (see Supplementary Figs. S1–S6).

**Figure 3:**
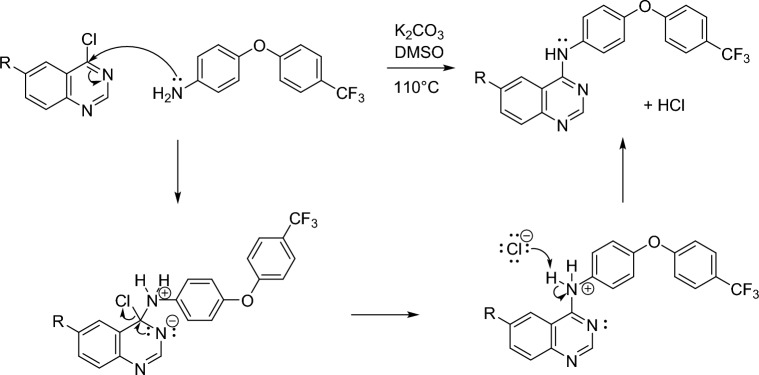
Proposed mechanism for the formation of ND-011992 (**1**) and bromine derivative (**2**).

This reaction constitutes a nucleophilic aromatic substitution (S_N_Ar) reaction, where the chlorine on the quinazoline reactant acts as a leaving group that is displaced by the nucleophilic aryl amine reactant. The proposed reaction mechanism is shown in Fig. 3.

### Inhibition of membrane bound enzyme complexes

**Figure 4:**
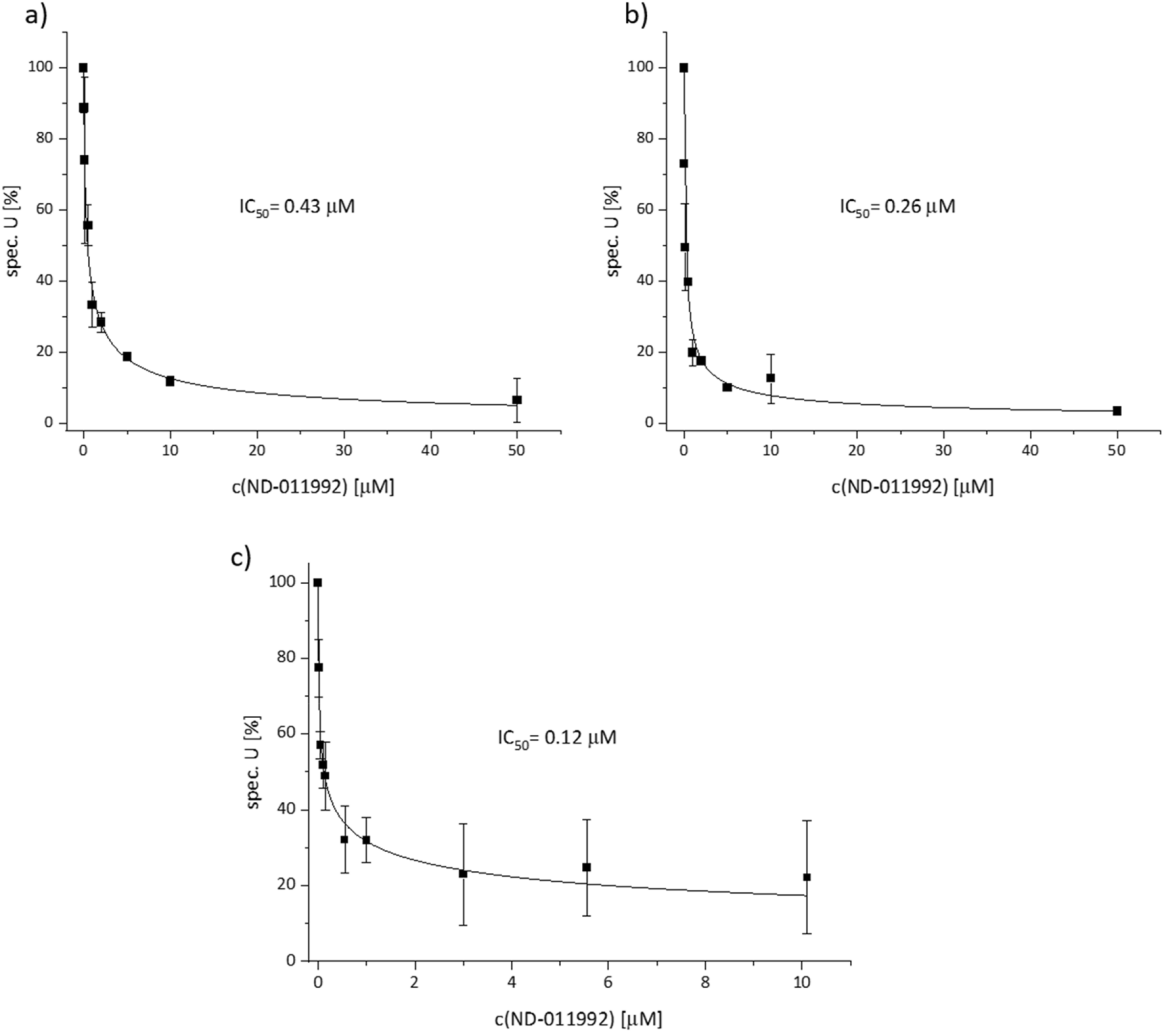
Oxidoreductase activities of *E. coli* BL21*Δ*cyo* membranes at various concentrations of ND-011992. (**a**) NADH oxidase activity, (**b**) d-NADH oxidase activity and (**c**) d-NADH:decyl-ubiquinone oxidoreductase activity of *E coli* CBO membranes plotted against concentration of ND-011992.

The inhibitory effect of ND-011992 on the NADH oxidase activity of membranes from *E. coli* strain BL21*Δ*cyo* was determined. This activity is mainly mediated by the primary NADH dehydrogenases and the terminal oxidases^13^. Strain BL21*Δ*cyo* chromosomally lacks the *bo*_3_-oxidase and contains the homologous *bd*-I and *bd*-II oxidases as the only terminal oxidases^14^. BL21*Δ*cyo* was grown in full medium, cells were harvested and disrupted and cytoplasmic membranes were prepared by differential centrifugation. With NADH as substrate, electrons might enter the respiratory chain either via complex I or via the alternative, non-energy converting NADH dehydrogenase, NDH-II. Titration of the NADH oxidase activity with ND-011992 revealed an IC_50_ of 0.43 µM (Fig. 4a). Second, deamino-NADH (d-NADH) was used as substrate to determine the d-NADH oxidase activity of BL21*Δ*cyo* membranes. d-NADH is a substrate specific for complex I preventing electron entry to the respiratory chain via NDH-II^15,16^. In this case, titration with ND-011992 revealed a significant lower IC_50_ of 0.26 µM (Fig. 4b). The shift of the IC_50_ in dependence from the substrate already indicates a high-affinity inhibition of respiratory complex I by ND-011992. To specifically monitor the inhibition of complex I, the d-NADH:decyl-ubiquinone oxidoreductase activity of cytoplasmic membranes of strain CBO was measured.

Strain CBO is chromosomally lacking both *bd* oxidases leaving *bo*_3_ oxidase as the only terminal oxidase^17^. The activity of *bo*_3_ oxidase was completely inhibited by an addition of 1 mM KCN to the assay^18^. Decyl-ubiquinone was added as external electron acceptor collecting electrons from the quinone-pool that is reduced by the reaction of complex I. With d-NADH as substrate, complex I is the only active enzyme in the assay. Here, the IC_50_ towards ND-011992 was determined to 0.12 µM (Fig. 4c). Thus, ND-011992 exhibits a sub-micromolar affinity towards complex I, making it a good inhibitor of this enzyme. The two-fold difference in the IC_50_ of ND-011992 in the d-NADH oxidase activities (Fig. 4b and c) might be due to differences between the two *E. coli* strains or to the additional contribution of the *bd* oxidases to the d-NADH oxidase activity. It can be concluded that ND-011992 is a good inhibitor of respiratory complex I in the bacterial membrane with an IC_50_ of 0.12 µM. To validate the claim that ND-011992 is a good and specific inhibitor of *bd* oxidases, we next investigated its inhibitory effect on the isolated oxidases.

### Inhibition of isolated quinol oxidases

*E. coli bd*-I oxidase was isolated from strain BL21*Δ*cyo*/pET28b( +)::*cydA*_*his*_*BX*^13^, *bd*-II oxidase from strain BL21*Δ*cyo*/pET28b( +)::*appC*_*his*_*BX*^19^ and *bo*_3_ oxidase from strain BL21*Δ*cyo*/pET28b( +)::*cyoA*_*his*_*BCD* (see ‘Methods’). The index “his” indicates the subunit of the enzyme complexes decorated with a His-tag. All strains were grown under oxic conditions in full medium. Cells were harvested and disrupted and cytoplasmic membranes were prepared by differential centrifugation. Membrane proteins were extracted with the detergent lauryl maltose neopentyl glycol (LMNG) and purified to homogeneity by affinity-, anion exchange- and size exclusion-chromatography (see Supplementary Figs. S7–S9).

**Figure 5:**
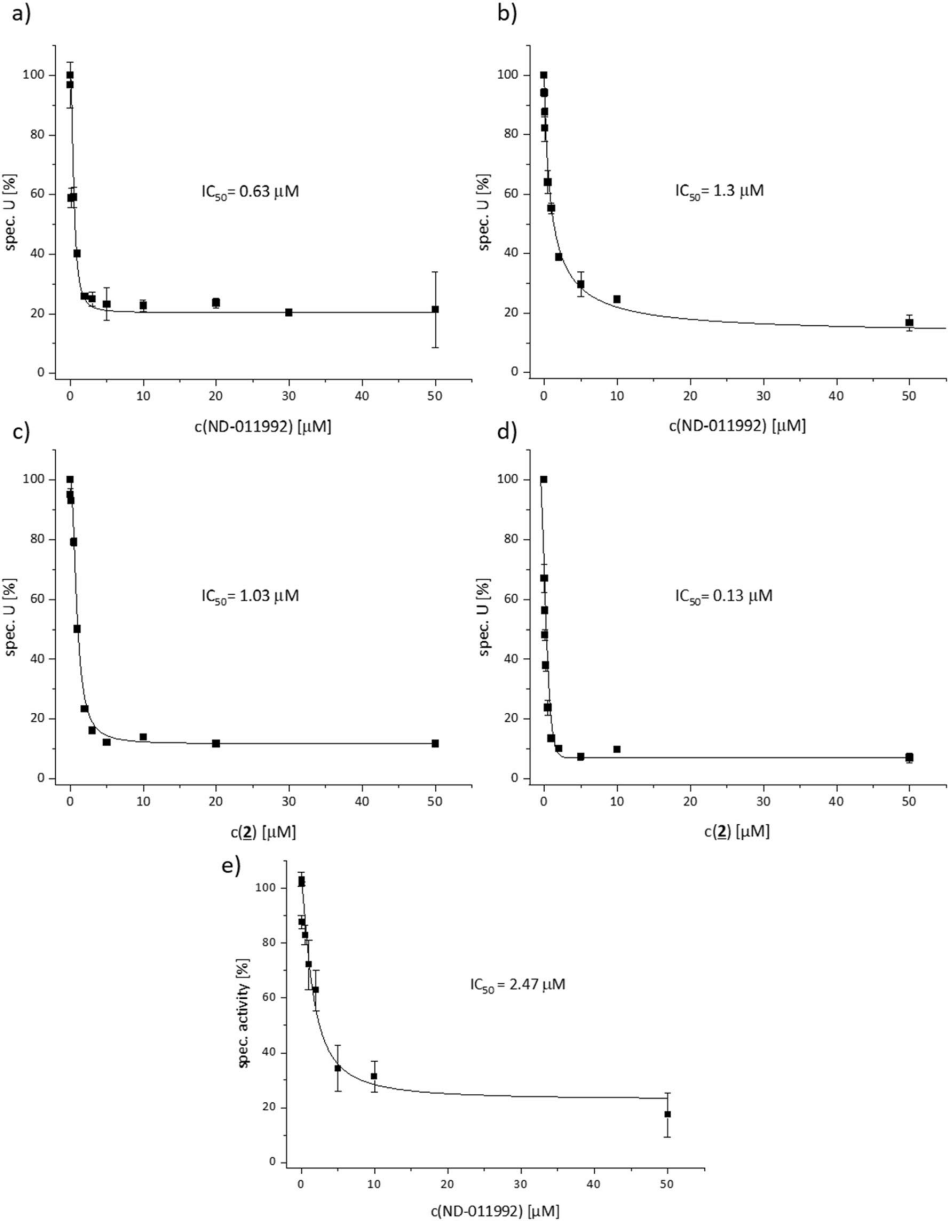
Duroquinol:oxygen oxidoreductase activity of isolated terminal *E. coli* oxidases at various concentrations of ND-011992 and compound **2**. (**a**) and (**b**) show the inhibition of *bd*-I and *bd*-II oxidases by ND-011992. (**c**) and (**d**) show the inhibition of *bd*-I and *bd*-II oxidases by **2**. Inhibition of *bo*_3_ oxidase with ND-011992 is shown in (**e**).

The affinity of ND-011992 towards the isolated oxidases was determined by its inhibition of the duroquinol:oxygen oxidoreductase activity measured as decrease of oxygen concentration^20^. The IC_50_ to *bd*-I and *bd*-II oxidase were determined to 0.63 and 1.3 µM, respectively (Fig. 5a and b). Thus, there is a significant two-fold difference in binding of ND-011992 towards the two homologous oxidases. Furthermore, the IC_50_ is higher than that to respiratory complex I, showing that the compound binds better to complex I than to *bd*-I and to *bd*-II oxidase. Incorporation of bromine to the compound led to significant changes of inhibition. The IC_50_ of the bromine derivative **2** to *bd*-I oxidase was slightly increased to 1.03 µM compared to that obtained with ND-011992. In contrast, the brominated compound showed a tenfold improved binding to *bd*-II oxidase with an IC_50_ of 0.13 µM (Fig. 5c and d). Surprisingly, **2** had no effect on the NADH oxidase activity of *E. coli* membranes in the micromolar concentration range. The most reasonable explanation is that **2** cannot enter the biological membrane due to the bulky bromine substitution and therefore does not encounter its target.

Because it was claimed that ND-011992 is an inhibitor specific for *bd* oxidases^9^, we also determined its IC_50_ towards the isolated *bo*_3_ oxidase. As *bd* oxidases, *bo*_3_ oxidase is also a quinol oxidase. Indeed, the IC_50_ of 2.47 µM is two- to four-fold higher than for the *bd* oxidases (Fig. 5e), however, all IC_50_ values are in a similar range.

In these experiments, all three oxidases are only partially inhibited, especially in the titration with ND-011992 about 20% residual activity remains (Fig. 5). Partial inhibition is found when an enzyme-inhibitor complex is still capable of producing a product. This clearly indicates that ND-011992 acts as a reversible inhibitor that does not bind to the exactly same site as the substrate but in close proximity. This is expected due to the distinctly different structures of the inhibitors and the substrate. In addition, the quinone binding sites of all the enzymes investigated have a completely different architecture (see ‘Discussion’), so that competitive inhibition to the respective substrate binding site is not to be expected.

**Figure 6:**
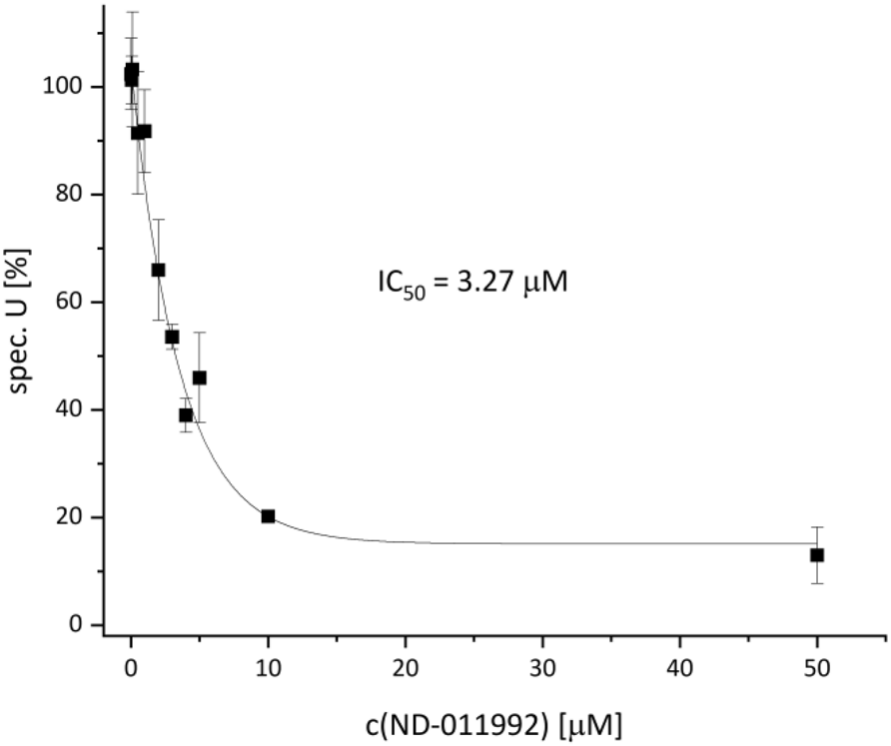
NADH oxidase activity of bovine mitochondrial membranes at various concentrations of ND-011992.

Inhibition of *E. coli* respiratory complex I by ND-011992 at sub-micromolar concentrations was unexpected. In order for ND-011992 or a derivative thereof to be used as a possible anti-tuberculosis drug, it must be ensured that the substance does not also inhibit mitochondrial complex I. To verify this, we investigated the inhibition of NADH oxidase activity of membranes from bovine heart mitochondria (Fig. 6). The rate-limiting step of this reaction cascade is respiratory complex I. The NADH oxidase activity was inhibited with an IC_50_ of 3.27 µM. Again, a residual activity of about 20% is observed. Inhibition of mitochondrial complex I is about 27-fold lower than that of bacterial complex I. This shows that ND-011992 is a suitable starting material to develop more effective and specific derivatives that specifically inhibit bacterial *bd* oxidases.

## Discussion

We investigated the effect of ND-011992, which has been described as an inhibitor of *bd* oxidases^9^, on various enzyme complexes of the *E. coli* aerobic respiratory chain. The IC_50_ values of ND-011992 to respiratory complex I, *bo*_3_ oxidase and both *E. coli bd* oxidases are all in the low micromolar range. Thus, ND-011992 is not a specific inhibitor that acts with high affinity on one target, but it inhibits quinone reductases and quinol oxidases of the respiratory chain. These enzyme complexes all have distinct differences in the architecture of their quinone binding sites. *E. coli* cytochrome *bo*_3_ ubiquinol oxidase contains a hydrophobic groove at the periplasmic side of the membrane. This quinol oxidation side is made up by one subunit^21,22^. The quinol headgroup interacts with conserved Asp, Arg and His residues, and the first five units of the isoprene side chain of the natural substrate ubiquinol-8 are clamped in a V-shaped form by three transmembranous helices. It was shown that the *bd* oxidases bind and oxidize ubiquinol with the help of a hydrophilic Q-loop domain that is positioned on the periplasmic side of the membrane^8,14,19,23–25^. This domain is located on top of the transmembranous part of the same subunit^26,27^. The exact position of bound ubiquinol is not yet known but the structure of *E. coli bd*-II oxidase with a bound quinol-site inhibitor, aurachin D, suggests binding of ubiquinol in a similar position^19^. The aurachin polar head group interacts with a conserved Asp residue and its hydrophobic chain is involved in several hydrophobic interactions with residues of the Q-loop and the transmembranous region of the same subunit. In contrast, the ubiquinone reduction site of respiratory complex I is made up by several subunits that form a large cavity extending from the hydrophobic membrane into the so-called peripheral arm of the complex I containing the cofactors of electron transfer. The ubiquinone headgroup is bound by conserved Tyr and His residues buried deep at the end of the cavity^28–30^. In the *E. coli* complex I, the isoprene side chain of ubiquinone-8 fills the entire cavity and faces several hydrophobic interactions^28^.

Due to the different structural arrangement of the quinone binding sites, it is reasonable to assume that ND-011992 mainly acts through its hydrophobicity and space filling structure to bind and block the access to the active sites. Remarkably, ND-011992 inhibits quinone reduction as found in respiratory complex I, and quinol oxidation, as present in quinol oxidases with similar efficacy. Thus, the compound exhibits a broad spectrum of putative targets. This makes it an interesting tool to e.g. efficiently shut down the entire respiratory chain. On the other hand it can be used as a starting point to develop more specific inhibitors. The molecule is freely available from one of the authors (DL) upon reasonable request.

Nevertheless, subtle differences were detectable in the inhibition of the various enzyme complexes of the respiratory chain. ND-011992 shows the best inhibitory power towards complex I with an IC_50_ of 0.12 µM (Fig. 4). The *bd* oxidases are inhibited with a five to eleven times lower efficacy. Remarkably, the IC_50_ to *bd*-I (0.63 µM) is twofold lower than to *bd*-II (1.3 µM). Furthermore, the bromine derivative **2** has a tenfold lower IC_50_ (0.13 µM) to *bd*-II than ND-011992 (Fig. 5). This might be due to the presence of a positively charged Lys residue in the large subunit of *bd*-II oxidase (K287) that is replaced by a neutral Gln residue in *bd*-I (Q287) in the putative Q-binding side of the *bd* oxidases. This makes this compound an attractive tool to study the quinol binding site of *bd*-II oxidase by cryo-electron microscopy.

Most interestingly, mitochondrial complex I is inhibited with a 27 times lower efficacy than *E. coli* complex I (IC_50_ = 0.12 vs. 3.27 µM). Furthermore, the inhibition of bacterial *bd* oxidases, which have no homologues in mitochondria, is still five to three times more effective than that of the mitochondrial complex. Thus, there is a good chance that further structural optimization of ND-011992 will lead to the development of even more specific and potent inhibitors. Lead optimization is also needed to improve the not so outstanding pharmacokinetic properties of ND-011992.

In light of the inhibitory results for ND-011992 (**1**) and bromine derivative **2** we conclude that these two molecules can be considered novel chemical probes for quinone/quinole converting enzymes due to their ability to entirely shut down respiration. With this work we are aiming to contribute to “target 2035”, an ambitious initiative by academic and pharmaceutical groups to develop small molecule probes for every protein in the proteome^31^.

## Methods

### General experimental and instrumentation for chemical synthesis

Commercially available reagents were purchased from Alfa Aesar, Millipore Sigma, and Acros Organics. Reaction setup included oven-dried glassware, magnetic stir plate and stir bar, a thermometer, and a heating mantle. Reactions were monitored by Thin-layer chromatography using Analtech Silica gel HLF UV254. TLC plates were visualized using a UV lamp. Flash chromatography was done with Acros Organics 0.035–0.070mm, 60 Å silica gel as the stationary phase.

ClogP data were obtained using the chemical properties tool in ChemDraw.

^1^H- and ^13^C-NMR data were taken on an Agilent VnmrJ4 400 MHz NMR Spectrometer and recorded in ppm against the NMR solvent (CDCl_3_) or TMS as the internal standard. Spectral data are reported in the following format: (1) ^1^H-NMR spectroscopy: chemical shifts (multiplicity, coupling constant, and number of protons); (2) ^13^C-NMR spectroscopy: chemical shift. Chemical shifts (δ) were reported in parts per million (ppm) and determined relative to the internal standard TMS (δ 0.0 ppm). Multiplicities were abbreviated as the follows: s = singlet, d = doublet, t = triplet, q = quartet, dd = doublets of doublet, m = multiplet, br = broad. GC/MS data were obtained using Agilent 6890N/5973 GCMS system.

### Synthesis of N-(4-(4-(trifluoromethyl)phenoxy)phenyl)quinazolin-4-amine (also referred to as ND-011992 (**1**))

4-Chloroquinazoline (CAS: 5190-68-1, 164 mg, 0.987 mmol), 4-(4-(trifluoromethyl)phenoxy)aniline (CAS: 57478–19-0, 256 mg, 0.987 mmol), and potassium carbonate (135 mg, 0.987 mmol) were dissolved in 7 mL of DMSO. The reaction was refluxed at 110 °C for 21 h. The reaction mixture was concentrated *in vacuo*, dissolved in DCM, and washed with 5% acetic acid solution (2x), water (1x), and brine (1x) and dried over sodium sulfate (Na_2_SO_4_). The organic layer was concentrated *in vacuo* to yield 0.381 g crude product (75% yield). The crude product was purified via silica gel flash chromatography using a 1:1 EA:DCM solvent system to yield 281 mg product collected as a white solid. Molecular weight: 381.36 g/mol. The hygroscopic purified product needed to be oven-dried at 110 °C overnight to evaporate water molecules seen in the proton NMR. Melting point range: 155.9–187.5 °C. ^**1**^**H-NMR** (400 MHz, CDCl_3_) δ ppm 8.791 (s, 1H), 7.958–7.939 (d, 1H), 7.906–7.886 (d, 1H), 7.857–7.816 (m, 1H), 7.798–7.759 (m, 2H), 7.620–7.577 (m, 3H), 7.432 (br. s, 1H), 7.151–7.077 (m, 3H). ^**13**^**C-NMR** (100 MHz, CDCl_3_) δ ppm 154.909, 152.260, 134.571, 134.571, 133.054, 133.054, 129.199, 127.173, 127.135, 126.785, 123.644, 120.624, 120.624, 120.077, 177.725, 177.725, 114.970. **EI-MS:** *m/z* 381.1 [M^+^] found at peak (retention time) from 27.691 to 29.548 min.

### Synthesis of 6-bromo-*N*-(4-(4-(trifluoromethyl)phenoxy)phenyl)quinazolin-4-amine (also referred to as bromine derivative (**2**))

6-Bromo-4-chloroquinazoline (CAS: 38267–96-8, 477 mg, 1.97 mmol), 4-(4-(trifluoromethyl)phenoxy)aniline (CAS: 57478–19-0, 502 mg, 1.97 mmol), and potassium carbonate (265 mg, 1.97 mmol) were dissolved in 7 mL of DMSO. The reaction was refluxed at 110 °C for 31 h. The reaction mixture was washed with 5% acetic acid solution (2 ×), water (1 ×), and brine (1 ×) and the organic layer concentrated *in vacuo*. The crude product was purified via silica gel flash chromatography using a 1:1 EA:DCM solvent system to yield 409 mg (45% yield) collected as a brown solid. Molecular weight: 460.25 g/mol. The hygroscopic purified product needed to be oven-dried at 110 °C for ~ 2 h 40 min. Melting point range: 206.7–207.7 °C. ^**1**^**H-NMR** (400 MHz, CDCl_3_) δ ppm 8.766 (s, 1H), 8.094–8.089 (d, 1H), 7.897–7.869 (dd, 1H), 7.821–7.799 (d, 1H), 7.758–7.735 (d, 2H), 7.599–7.577 (d, 2H), 7.528 (br. s, 1H), 7.137–7.074 (m, 4H). ^**13**^**C-NMR** (100 MHz, CDCl_3_) δ ppm 206.966, 156.889, 155.076, 152.268, 148.640, 136.210, 134.693, 130.367, 127.142, 127.104, 127.066, 127.028, 123.909, 120.366, 119.743, 117.725.

### Strains and cell growth

Cytoplasmic membranes from *E. coli* strain BL21*Δ*cyo* that chromosomally lacks the *cyoABCD* genes encoding cytochrome *bo*_3_ oxidase and strain CBO that chromosomally lacks the *cydABX* and *appCBX* genes encoding the cytochrome *bd*-I and *bd*-II oxidase, respectively, were used to determine enzyme activity in the membrane^13,17,19^. Cytochrome *bo*_3_ ubiquinol oxidase was isolated from *E. coli* strain BL21*Δ*cyo*/pET28b( +)::*cyoA*_*his*_*BCD* with the genes encoding the oxidase on an expression plasmid. Cytochrome *bd*-I ubiquinol oxidase was isolated from strain BL21*Δ*cyo*/pET28b( +)::*cydA*_*his*_*BX* with the genes encoding the *bd*-I oxidase on an expression plasmid^14^. Cytochrome *bd*-II ubiquinol oxidase was isolated from strain BL21*Δ*cyo*/pET28b( +)::*appC*_*his*_*BX* with the genes encoding the *bd*-II oxidase on an expression plasmid^19^. Cell cultures were grown aerobically in 2 L baffled flasks each containing 800 mL LB-medium at 37 °C. Gene expression was induced at an OD_600_ of approximately 2 by the addition of 400 µM isopropyl β-D-1-thiogalacto-pyranoside. Cells were harvested 2 h after induction by centrifugation and stored at -80 °C^19^.

### Preparation of cytoplasmic membranes

All steps were carried out at 4 °C. 40 g shock frozen cells (wet weight) were homogenized in a six-fold volume of 20 mM 3-(N-morpholino)propanesulfonic acid (MOPS), 20 mM NaCl, 0.5 mM phenylmethylsulfonylfluoride (PMSF), pH 7.0 and a few grains of DNase I in a teflon-in-glass homogenizer. Cells were disrupted with three cycles in a high pressure homogenizer (1250 bar, Maximator HPL6, Maximator GmbH) and cell debris was removed by centrifugation (4 °C, 10′000 rpm, 30′000 × g; 20 min, Rotor 25.50, Avanti J-26S XP). Membranes were obtained from the supernatant by ultra-centrifugation (4 °C, 50′000 rpm, 250′000 × *g*, 70 min, Rotor 60Ti, Optima LE80-K, Beckman-Coulter). The sedimented membranes were re-suspended in a 1:1 ratio (w/v) with 20 mM MOPS and 20 mM NaCl, pH 7.0 in a teflon-in-glass homogenizer.

### Protein extraction and purification

Preparation of cytochrome *bd*-I and cytochrome *bd*-II ubiquinol oxidase from *E. coli* was described previously^13,19^. For the purification of *E. coli* cytochrome *bd*-I ubiquinol oxidase, membrane proteins were extracted with the detergent LMNG and loaded on a Probond Ni^2+^-IDA column. Proteins were eluted from the washed column by a linear imidazole gradient. The pooled peak fractions were concentrated and applied onto a ResourceQ ion exchange column in a NaCl gradient. The concentrated samples were applied onto a HiPrep 16/60 Sephacryl S-300 HR size-exclusion chromatography column. Peak fractions were pooled and stored at a final concentration of 2 mg/ml in 20 mM MOPS, 20 mM NaCl, 0.003% LMNG, pH 7.0^13^. For the purification of *E. coli* cytochrome *bd*-II ubiquinol oxidase, membrane proteins were extracted with LMNG and loaded on a Probond Ni^2+^-IDA column. Proteins were eluted from the washed column with 260 mM imidazole. The concentrated samples were applied onto a HiLoad 16/60 Superdex 200 pg size-exclusion chromatography column. Peak fractions were pooled and stored at a final concentration of 2 mg/ml in 20 mM MOPS, 20 mM NaCl, 0.003% LMNG, pH 7.0^19^. For the purification of *E. coli* cytochrome *bo*_3_ ubiquinol oxidase, membrane proteins were solubilized with 2% (w/v) LMNG for 60 min at 4 °C. After centrifugation (4 °C, 50′000 rpm, 250′000 × *g*, 15 min, Rotor 60Ti, Optima LE80-K, Beckman-Coulter), the supernatant was loaded on a Ni^2+^ affinity chromatography column (ProBond, 25 mL, Life Technologies) equilibrated in 50 mM MOPS, 500 mM NaCl, 50 mM imidazole, 0.5 mM PMSF, 0.005% LMNG, pH 7.5. The column was washed with the same buffer containing 92 mM imidazole. Bound proteins were eluted with 284 mM imidazole. Peak fractions were pooled and concentrated to 0.5 mL. The concentrated protein was applied to size-exclusion chromatography on a Superose 6 column (GE Healthcare) equilibrated in 50 mM MOPS, 20 mM NaCl, 0.5 mM PMSF, 0.005% LMNG, pH 7.5. Fractions containing the *bo*_3_ oxidase were pooled and concentrated to 20 mg/mL.

### Activity measurements

The (d-)NADH oxidase activity of cytoplasmic membranes was determined with an Clark-type oxygen electrode (Oxyview System, Hansatech Instruments) at 30 °C^19^. 5 µL membrane suspensions (50 mg/mL) were added to 2 mL 20 mM MOPS, 20 mM NaCl, pH 7.0. The non-enzymatic reaction was determined after formation of a stable baseline. The reaction was started by an addition of 5 µL (d-)NADH (0.5 M) and the catalytic rates were corrected for the non-enzymatic reaction. After 1 min, the reaction was inhibited by adding various amounts (0.01–50 µM) ND-011992. Each data point was assayed in triplicates. The protein concentration of membranes were determined by the biuret-method^32^. The standard deviation of each measurement is provided.

The d-NADH:decyl-ubiquinone oxidoreductase activity of cytoplasmic membranes was measured as decrease of the d-NADH concentration at 340 nm using an ε of 6.3 mM^−1^ cm^−1^ (QS cuvette, d = 1 cm, Hellma; TIDAS S500, J&M Aalen) ^15^. 10 µL membrane suspension (100 mg/mL) were added to 1 mL 50 mM MES/NaOH, 50 mM NaCl, 5 mM MgCl_2_, pH 6.0. The activity of the *bo*_3_ oxidase was inhibited by an addition of 1 mM KCN. 150 μM d-NADH was added after 30 s incubation. The reaction was started after 1 min by an addition of 400 μM decyl-ubiquinone. The rates were corrected for the non-enzymatic reaction. After 1 min, the reaction was inhibited by adding various amounts (0.01–50 µM) of ND-011992. Each data point was assayed in triplicates. The standard deviation of each measurement is provided.

The duroquinol:oxygen oxidoreductase activity of isolated *E. coli bd*-I, *bd*-II and *bo*_3_ oxidase was determined by monitoring the oxygen consumption with a Clark-type oxygen electrode (Oxyview System, Hansatech Instruments)^20,33^. All steps were conducted at 30 °C. The reaction chamber was filled with 2 mL 20 mM MOPS, 20 mM NaCl, 0.003% LMNG, pH 7.0. After formation of a stable baseline, 10 μL dithiothreitol (DTT) (1 M) and 5 μL duroquinone in ethanol (100 mM) were added and incubated for 1 min. The non-enzymatic reaction rate was determined. 10 μL enzyme in LMNG (2 mg/mL) were added to start the reaction. The rates were corrected for the non-enzymatic reaction. After 1 min, the reaction was inhibited by adding various amounts (0.01–50 µM) of ND-011992 and **2**, respectively. Each data point was measured in triplicates. The standard deviation of each measurement is provided.

The NADH oxidase activity of isolated mitochondria from bovine heart was determined with a Clark-type oxygen electrode at 30 °C^34^. 50 µL mitochondrial membranes in 2 mL 0.44 M sucrose, 20 mM Tris, 2 mM EDTA, pH 7.4 were placed in the reaction chamber. After formation of a stable baseline, 20 µL NADH (0.5 M) was added to start the reaction. After 1 min, the reaction was inhibited by adding various amounts (0.01–50 µM) ND-011992. Each data point was assayed in triplicates. The standard deviation of each measurement is provided.

## Supplementary Information

Supplementary Information.

## Data Availability

The source data underlying Figs. 4, 5, 6 and other data are available from the corresponding authors upon reasonable request.
