# Supplementary material for: Exploring ND-011992, a quinazoline-type inhibitor targeting quinone reductases and quinol oxidases
Source: Sci Rep. 2023 Jul 28;13:12226. doi: 10.1038/s41598-023-39430-w (PMC10382516; doi:10.1038/s41598-023-39430-w)
Supplement: Supplementary file 1 — Supplementary Information. [file 41598_2023_39430_MOESM1_ESM.docx]

Supplementary Information

**Exploring ND-011992, a quinazoline-type inhibitor targeting quinone reductases and quinol oxidases**

Jan Kägi, Willough Sloan, Johannes Schimpf, Hamid R. Nasiri, Dana Lashley, and Thorsten Friedrich

**Spectral characterization of compounds**

**N-(4-(4-(trifluoromethyl)phenoxy)phenyl)quinazolin-4-amine (1)**

**^1^H-NMR** (400 MHz, CDCl_3_) δ ppm 8.779 (s, 1H), 7.954-7.931 (dd, J = 7.2, 1.2 Hz, 2H), 7.845-7.761 (m, 3H), 7.641 (br. s, 1H), 7.604-7.562 (m, 3H), 7.132 – 7.073 (m, 4H).

**^13^C-NMR** (100 MHz, CDCl_3_) δ ppm 160.51, 157.64, 154.78, 152.31, 149.71, 134.55, 133.12, 128.70, 127.13 (q, J = 3.8 Hz), 126.77, 125.12 (q, J = 32 Hz), 123.87, 122.81, 120.53, 120.48, 117.74, 115.01.

**EI-MS:** *m/z* 381.1 [M^+^] found at peak (retention time) from 27.691-29.548 min.


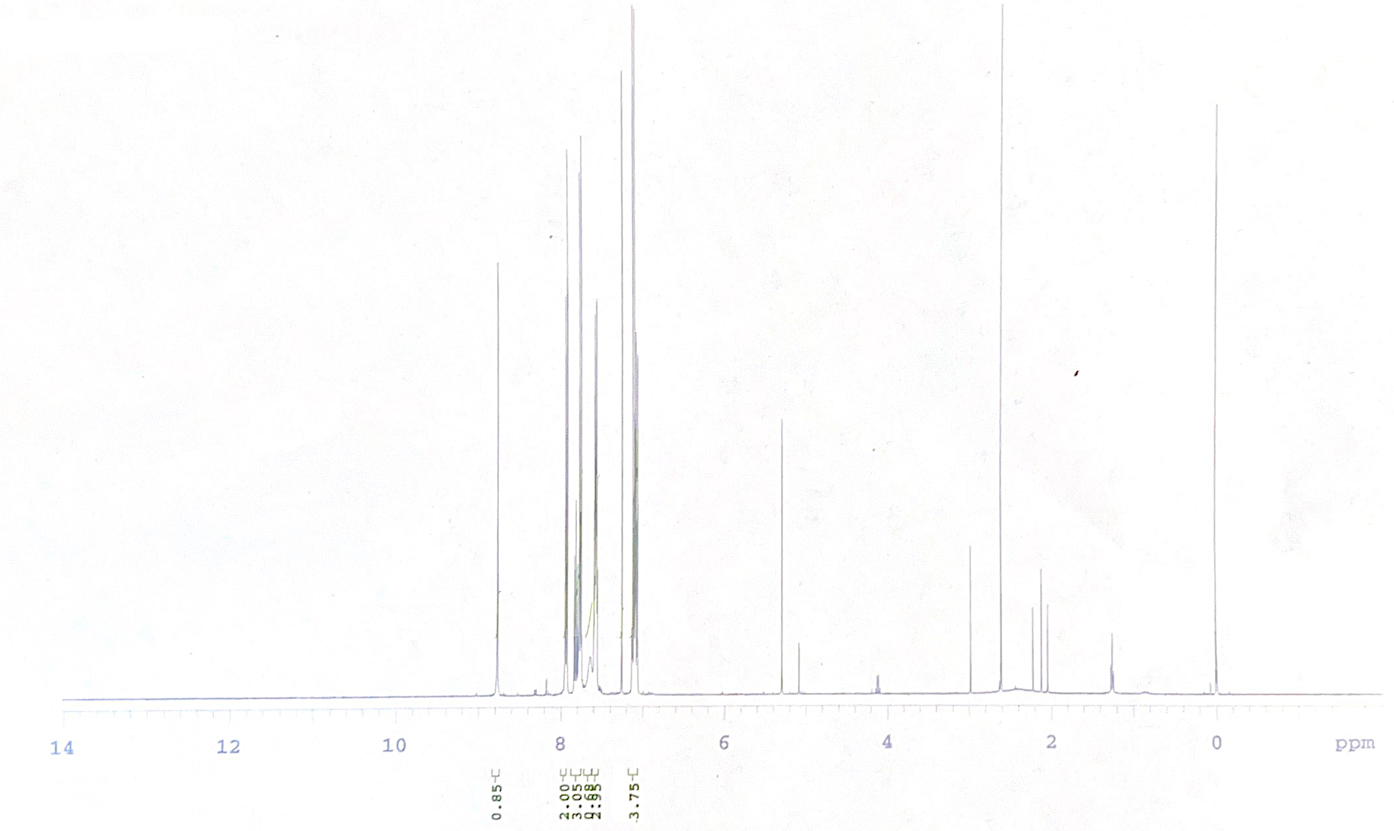


**Supplementary Figure 1.** ^1^H-NMR spectrum of **1**.


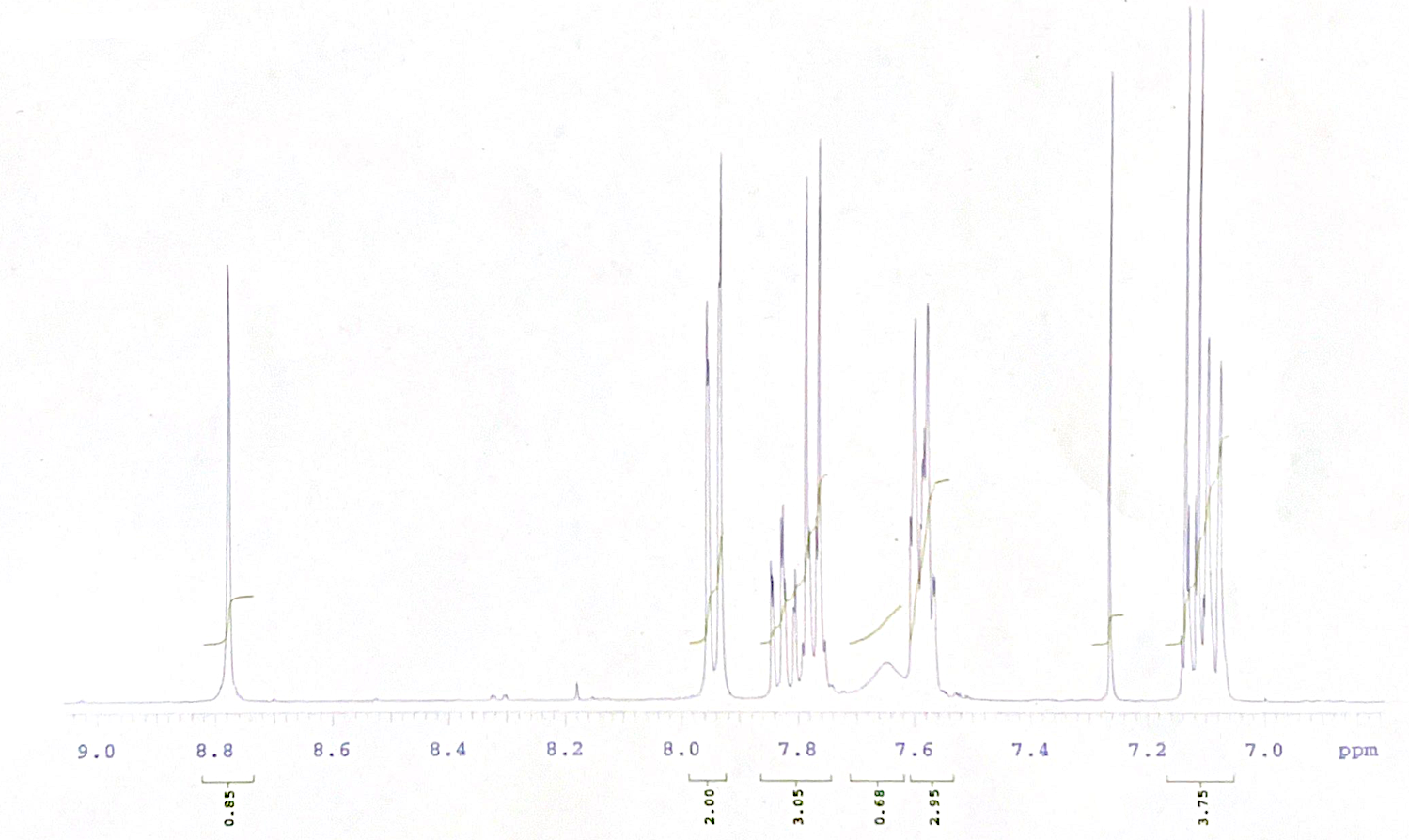


**Supplementary Figure 2.** Zoom into the aromatic region of the ^1^H-NMR of **1**.


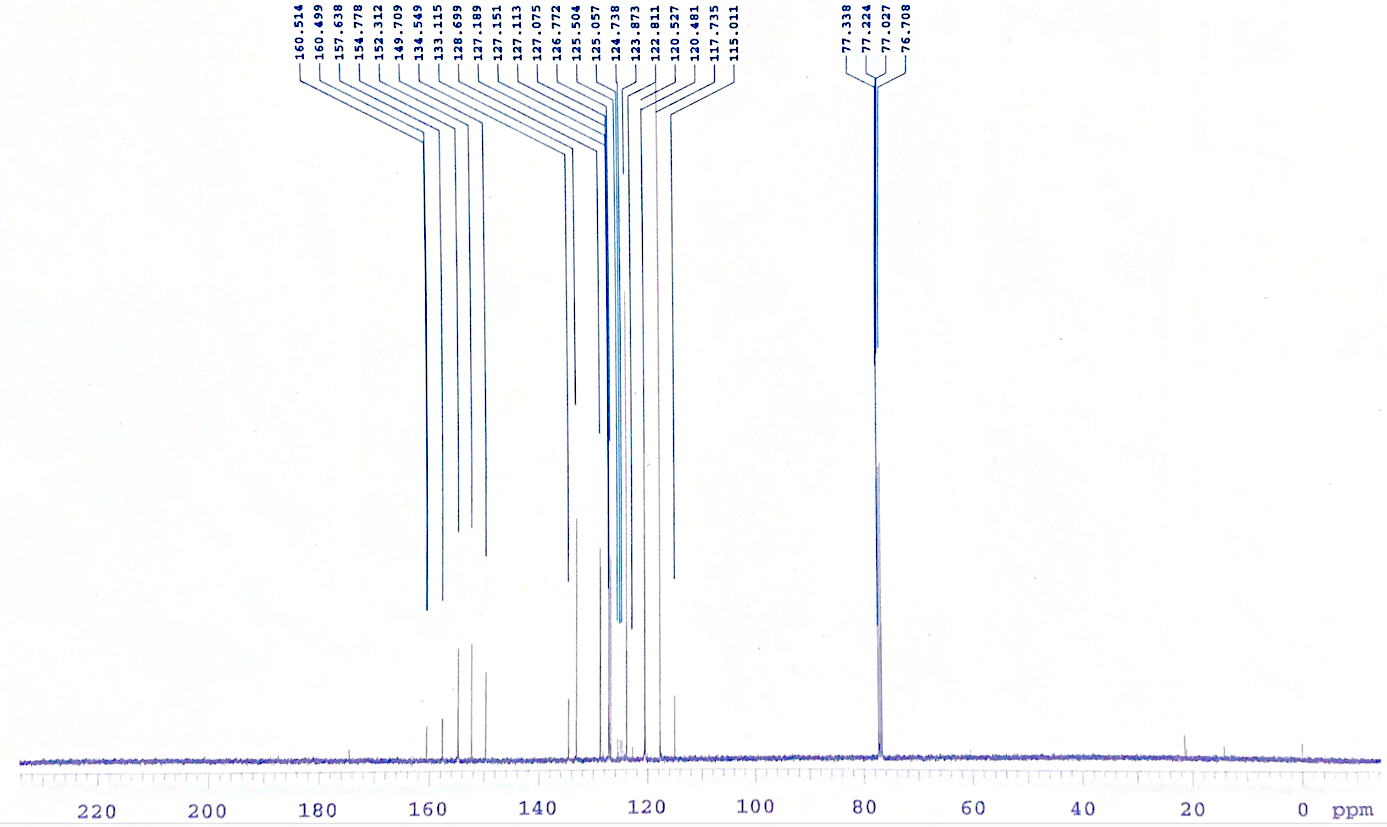


**Supplementary Figure 3.** ^13^C-NMR spectrum of **1**.

**N-(4-(4-(trifluoromethyl)phenoxy)phenyl)quinazolin-4-amine (2)**

**^1^H-NMR** (400 MHz, CDCl_3_) δ ppm 8.766 (s, 1H), 8.094-8.089 (d, 1H), 7.897-7.869 (dd, 1H), 7.821-7.799 (d, 1H), 7.758-7.735 (d, 2H), 7.599-7.577 (d, 2H), 7.528 (br. s, 1H), 7.137-7.074 (m, 4H).

**^13^C-NMR** (100 MHz, CDCl_3_) δ ppm 206.966, 156.889, 155.076, 152.268, 148.640, 136.210, 134.693, 130.367, 127.142, 127.104, 127.066, 127.028, 123.909, 120.366, 119.743, 117.725.


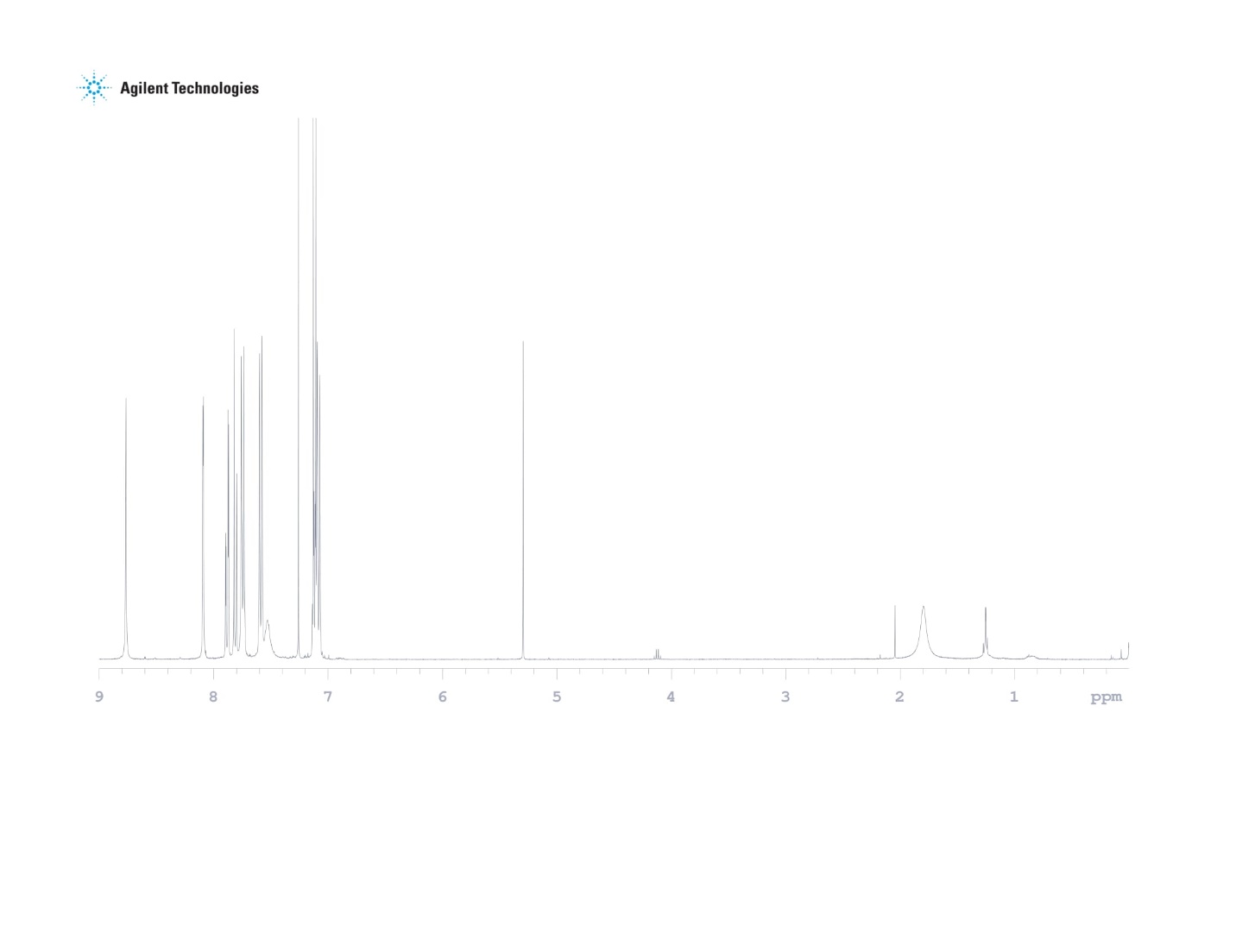


**Supplementary Figure 4.** ^1^H-NMR spectrum of **2**.


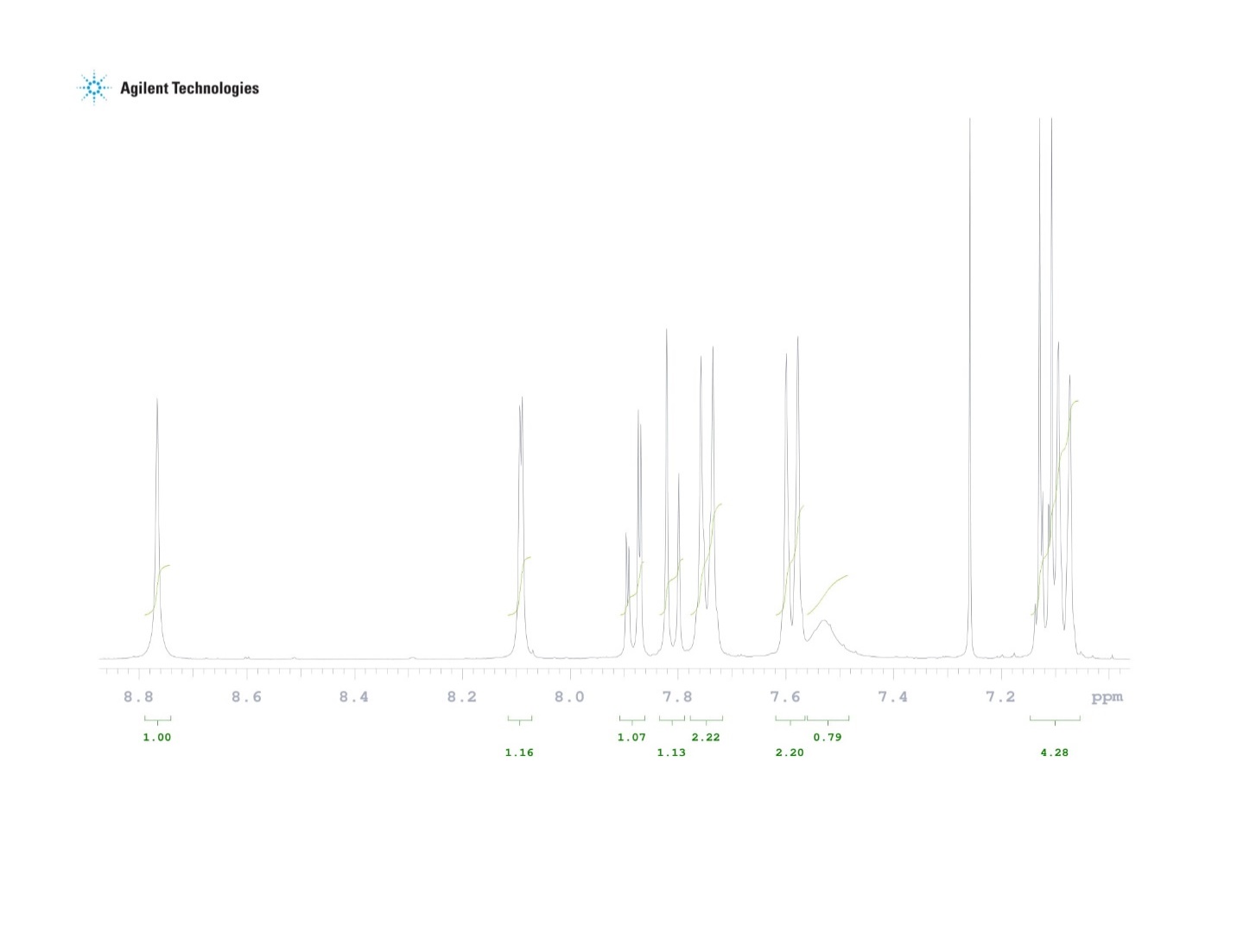


**Supplementary Figure 5.** Zoom into the aromatic region of the ^1^H-NMR of **2**.


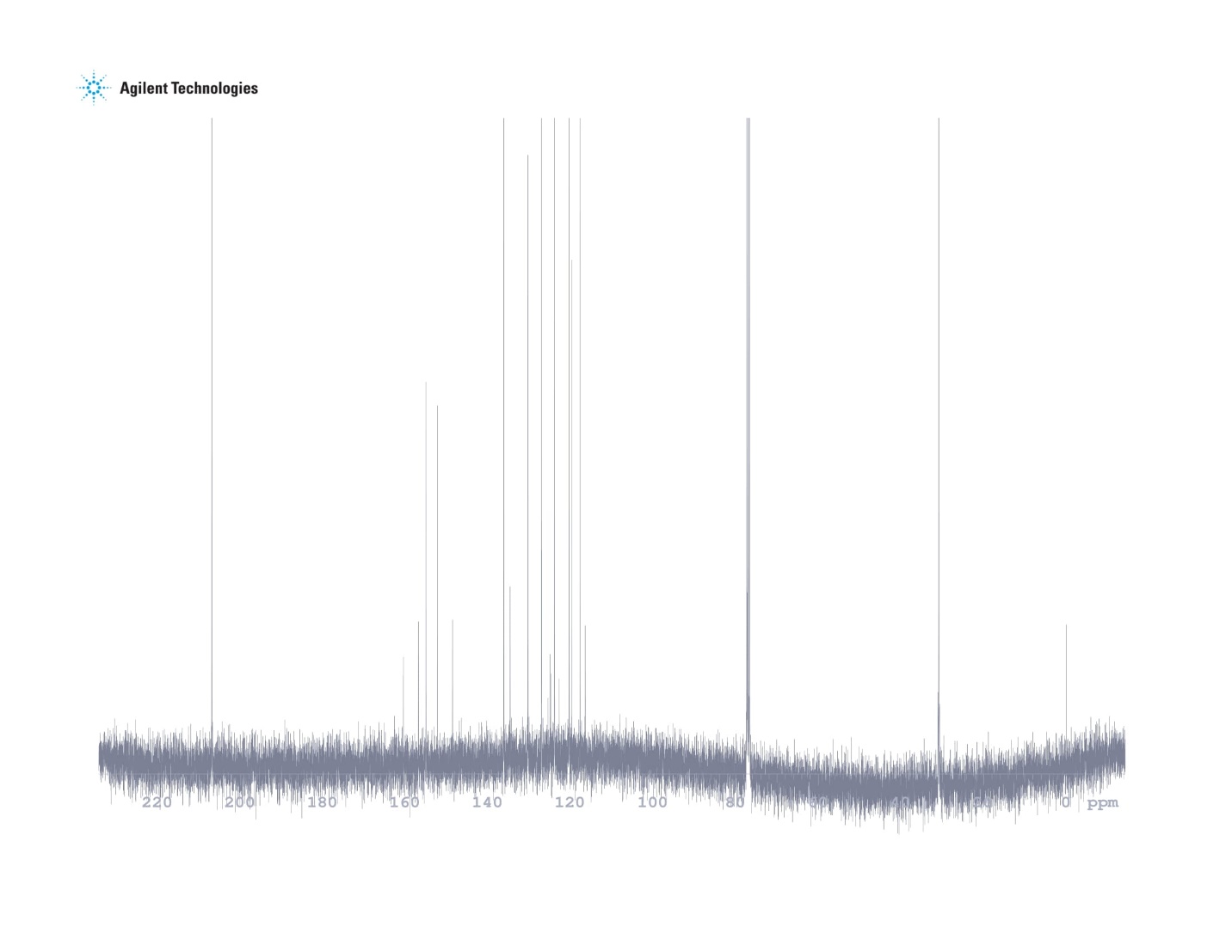


**Supplementary Figure 6.** ^13^C-NMR spectrum of **2**.


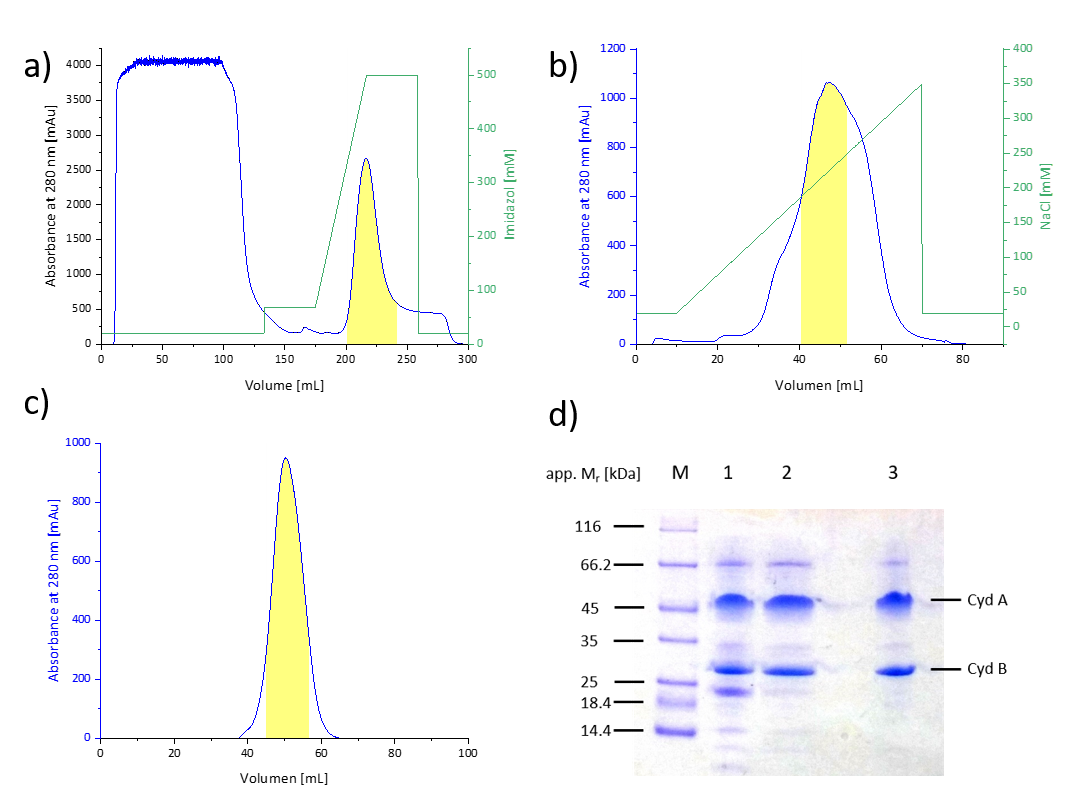


e)


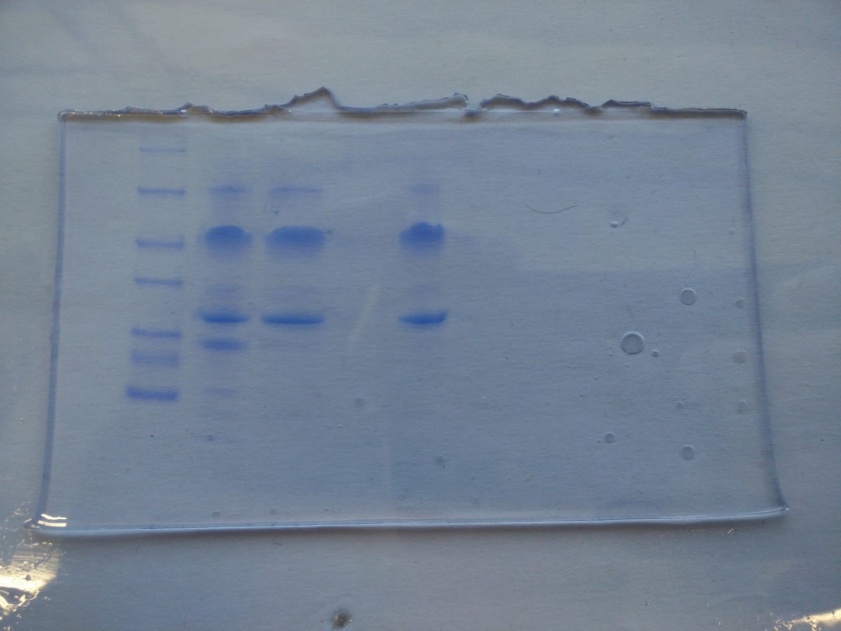


**Supplementary Figure 7. Preparation of cytochrome *bd-*I ubiquinol oxidase from *E. coli* strain BL21*Δ*cyo*/pET28b(+)::*cydAhBX*.** Elution profiles from a) Probond Ni^2+^-IDA, b) ResourceQ and from c) HiPrep 16/60 Sephacryl S-300 HR. The absorbance at 280 nm is shown in blue, the imidazole and NaCl concentrations in green. The pooled fractions are indicated by the yellow area. d) SDS-PAGE of the preparation. Lane 1 shows the protein pattern of the eluate from affinity chromatography, lane 2 that of the eluate from anion-exchange chromatography and lane 3 the eluate from the size-exclusion chromatography on a 10% gel after Coomassie Blue staining. Lane M shows the pattern of marker proteins. e) shows the complete gel shown in detail in d).


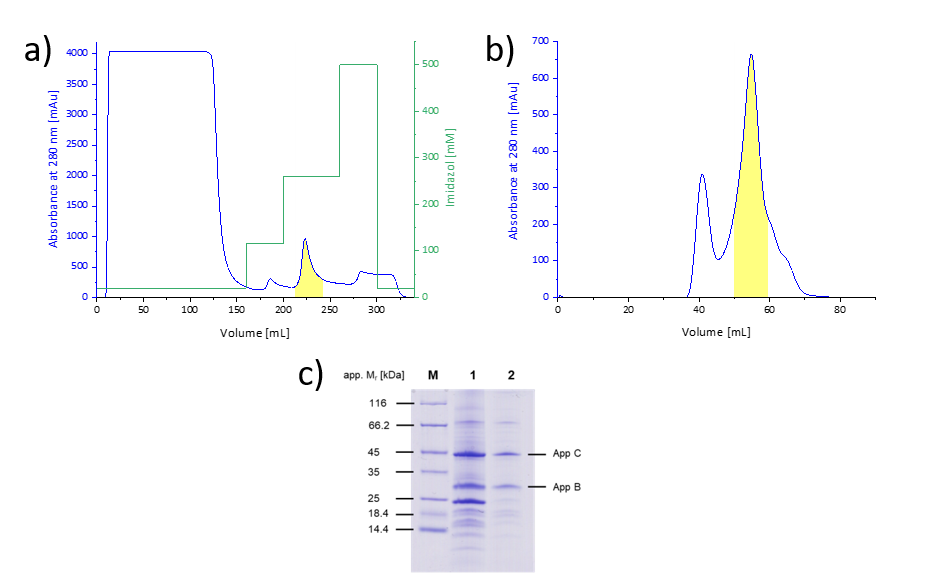


d)


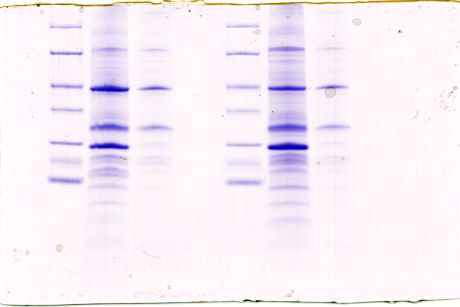


**Supplementary Figure 8. Preparation of cytochrome *bd-*II ubiquinol oxidase from *E. coli* strain BL21*Δ*cyo*/pET28b(+)::*appChBX*.** Elution profiles from a) Probond Ni^2+^-IDA and b) HiLoad 16/60 Superdex 200 pg. The absorbance at 280 nm is shown in blue, the imidazole concentration in green. The pooled fractions are indicated by the yellow area. c) SDS-PAGE of the preparation. Lane 1 shows the protein pattern of the eluate from affinity chromatography and lane 2 that of the eluate from size-exclusion chromatography on a 10% gel after Coomassie Blue staining. Lane M shows the pattern of marker proteins. d) shows the complete gel shown in detail in c).


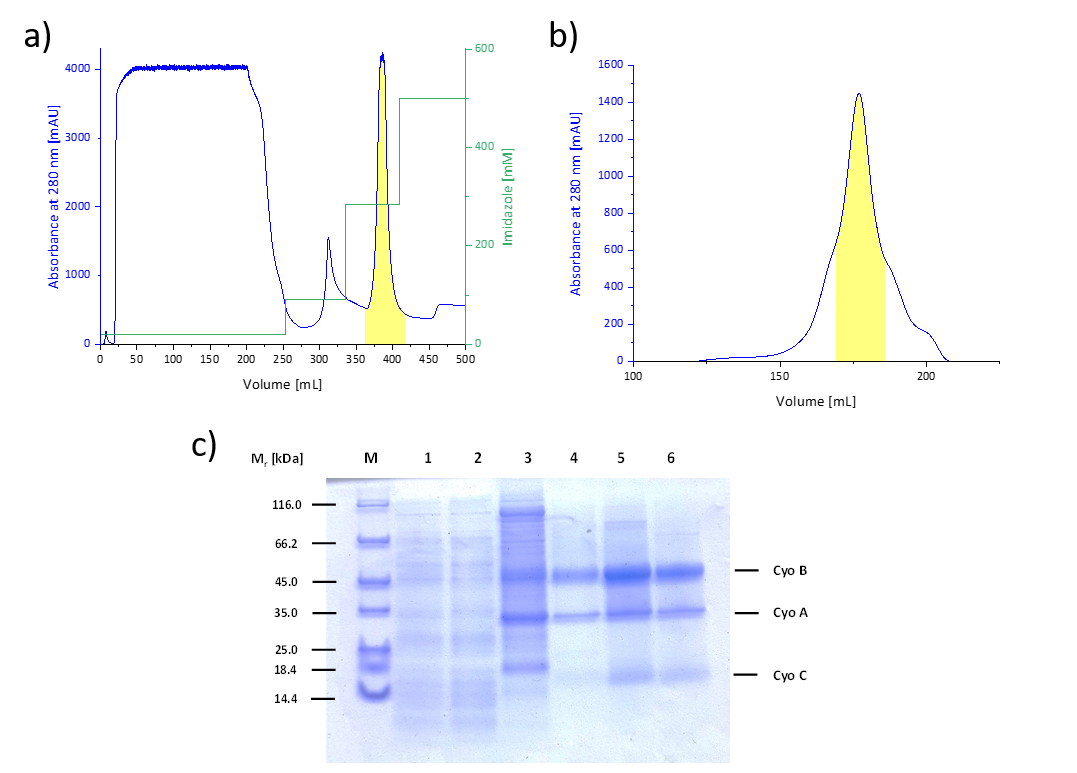


d)


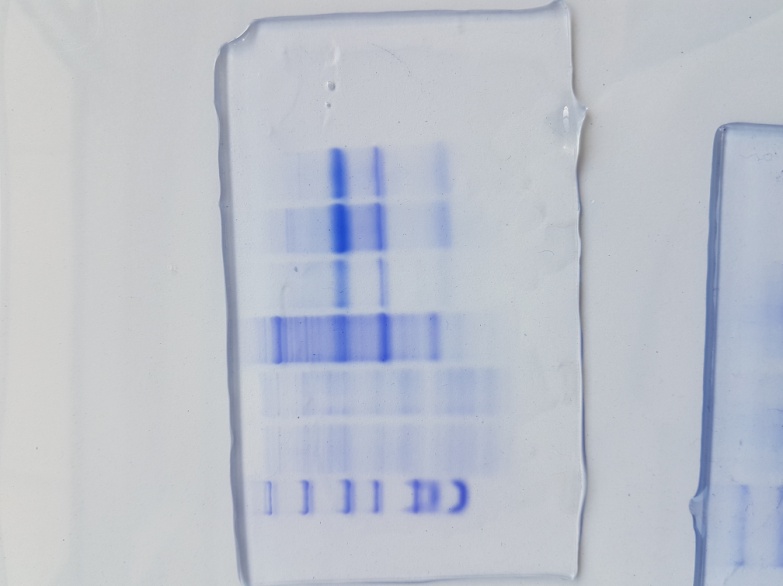


**Supplementary Figure 9. Preparation of cytochrome *bo*_3_ ubiquinol oxidase from *E. coli* BL21*Δ*cyo*/pET28b(+)::*cyoA_his_BCD*.** Elution profiles from a) Probond Ni^2+^-IDA and b) Superose 6. The absorbance at 280 nm is shown in blue, the imidazole concentration in green. The pooled fractions are indicated by the yellow area. c) SDS-PAGE of the preparation. Lane 4 shows the protein pattern of the eluate from affinity and lane 6 that of the eluate from size-exclusion chromatography on a 10% gel after Coomassie Blue staining. Lane M shows the pattern of marker proteins. Lanes 1 and 2 show the pattern of the through-flow of the affinity column and lane 3 that of the first peak after affinity chromatography. d) shows the complete gel shown in detail in c).
